# Development and evaluation of a novel Vital Signs Alert device for use in pregnancy in low-resource settings

**DOI:** 10.1136/bmjinnov-2017-000235

**Published:** 2018-09-19

**Authors:** Hannah L Nathan, Nicola Vousden, Elodie Lawley, Annemarie de Greeff, Natasha L Hezelgrave, Nicola Sloan, Nina Tanna, Shivaprasad S Goudar, Muchabayiwa F Gidiri, Jane Sandall, Lucy C Chappell, Andrew H Shennan

**Affiliations:** 1 Department of Women and Children’s Health, King’s College London, London, UK; 2 Validate Global, Kimberley, South Africa; 3 JNMC Women’s and Children’s Health Research Unit, KLE University, Belagavi, India; 4 College of Health Sciences, University of Zimbabwe, Harare, Zimbabwe

**Keywords:** affordable, diagnostics, global Health, obstetrics, hypertension

## Abstract

**Objectives:**

Haemorrhage, hypertension, sepsis and abortion complications (often from haemorrhage or sepsis) contribute to 60% of all maternal deaths. Each is associated with vital signs (blood pressure (BP) and pulse) abnormalities, and the majority of deaths are preventable through simple and timely intervention. This paper presents the development and evaluation of the CRADLE Vital Signs Alert (VSA), an accurate, low-cost and easy-to-use device measuring BP and pulse with an integrated traffic light early warning system. The VSA was designed to be used by all cadres of healthcare providers for pregnant women in low-resource settings with the aim to prevent avoidable maternal mortality and morbidity.

**Methods:**

The development and the mixed-methods clinical evaluation of the VSA are described.

**Results:**

Preliminary fieldwork identified that introduction of BP devices to rural clinics improved antenatal surveillance of BP in pregnant women. The aesthetics of the integrated traffic light system were developed through iterative qualitative evaluation. The traffic lights trigger according to evidence-based vital sign thresholds in hypertension and haemodynamic compromise from haemorrhage and sepsis. The VSA can be reliably used as an auscultatory device, as well as its primary semiautomated function, and is suitable as a self-monitor used by pregnant women.

**Conclusion:**

The VSA is an accurate device incorporating an evidence-based traffic light early warning system. It is designed to ensure suitability for healthcare providers with limited training and may improve care for women in pregnancy, childbirth and in the postnatal period.

## Introduction

WHO data from 2015 state that the lifetime risk of maternal death in high-income countries is 1 per 6000 pregnancies compared with 1 in 36 in Sub-Saharan Africa.^1^ Efforts to rectify this stark inequity is a global health priority. Identifying mechanisms to achieve this is the challenge.

Approximately 800 women die each day from complications of pregnancy and childbirth.^2^ Obstetric haemorrhage, hypertensive disorders, sepsis and abortion complications (often from haemorrhage or sepsis) contribute to 60% of all maternal deaths. Each of these conditions is associated with abnormalities in blood pressure and pulse.^2^ The majority of maternal deaths are preventable through simple intervention if administered promptly, including induction of labour, antihypertensives, misoprostol, uterine balloon tamponade and antibiotics.^3^ These interventions are often available even in the most low-resource settings; however, a common barrier to prompt intervention is a failure to recognise the severity of the condition.^4 5^

Detection of abnormal vital signs, including blood pressure and pulse, allows early recognition of women who require urgent treatment or transfer to higher intensity of care, and is critical in preventing mortality and morbidity. Despite the universally recognised importance of blood pressure and pulse measurement, access to accurate blood pressure devices, healthcare provider training on measurement and adequate escalation pathways are lacking for many women in low-income and middle-income countries (LMICs).^6^

Even in high-income countries, it is acknowledged that failures by healthcare providers to immediately recognise and act on signs of life-threatening conditions, particularly haemorrhage, pre-eclampsia and sepsis, contribute to avoidable maternal deaths.^7 8^ To aid earlier recognition of compromise, modified obstetric early warning system charts are now encouraged internationally for use in the care of pregnant and postpartum women.^7–11^ However, these charts are based on limited evidence, are labour-intensive and require clinician interpretation.

In this article, we report the progress of the CRADLE programme: the development and evaluation of the CRADLE Vital Signs Alert (VSA) device, a low-cost, accurate and easy-to-use vital signs measuring device developed over the past 15 years. It is a hand-held, upper-arm, semiautomated device developed in collaboration with Microlife, with the primary goal of ensuring accuracy and applicability in pregnancy.

### Ensuring accuracy

In 2006, we validated the device for use in a non-pregnant adult population, according to a formal validation protocol for assessing accuracy of automated devices.^12^ The cohort included participants with a range of ages, gender distribution, arm circumferences and blood pressure values, as stipulated by the protocol. For each participant, the validation process involved multiple measurements of blood pressure by trained observers under test conditions, alternating between the test device and two calibrated mercury sphygmomanometers. Assessment of accuracy was determined by comparing the mean difference (SD) between measurements from the test device and the mercury sphygmomanometers.

Validation in non-pregnant adults does not imply accuracy in pregnant women. Following the British Hypertension Society (BHS) guidelines,^13^ in 2012 we successfully validated the device in pregnancy, including women with pre-eclampsia.^14^ Our device is one of only a few devices suitable for non-pregnant and pregnant adults. Uniquely, we have also validated the device in pregnant women with low blood pressure (in South Africa, a LMIC), demonstrating the device maintains accuracy at the lower end of the range.^15^

### Creation of the VSA device

**Figure 1:**
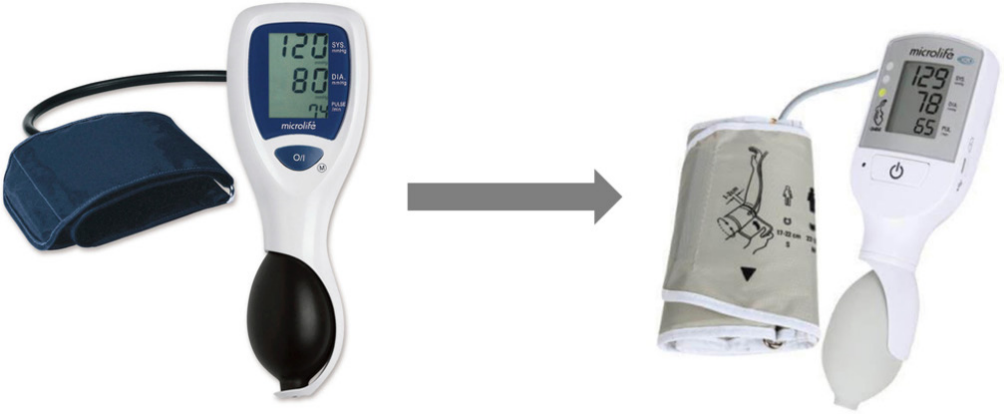
The prototype Microlife 3AS1-2 and the final CRADLE Vital Signs Alert, with traffic light early warning system.

**Figure 2:**
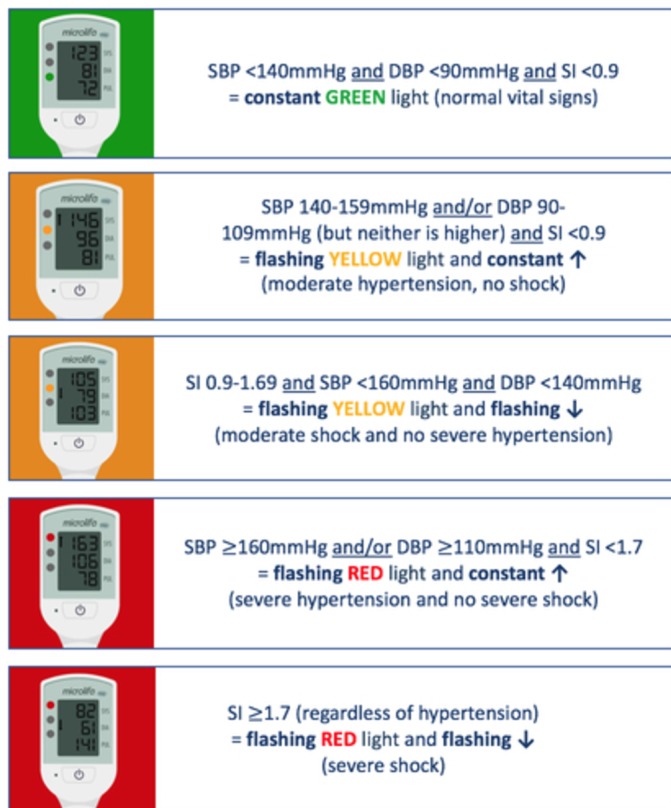
Vital Signs Alert device traffic light early warning system display options. DBP, diastolic blood pressure; SBP, systolic blood pressure; SI, Shock Index.

Through Bill & Melinda Gates Foundation funding, we modified the VSA device to ensure suitability for community healthcare providers in LMICs. It now meets the WHO’s requirements, being affordable (US$20), robust, easy-to-use, portable, requiring infrequent calibration and has low power requirements (charging through a micro-USB port) and dual auscultatory/oscillometric function.^14 16^ We incorporated a traffic light early warning system, aiming to alert untrained healthcare providers to vital sign abnormalities from hypertension, or shock secondary to obstetric haemorrhage and sepsis (figure 1). For maternal hypertension, established blood pressure thresholds trigger the green, yellow and red traffic lights of the early warning system. For shock (from haemorrhage or sepsis), we determined the early warning system thresholds through retrospective analysis of vital signs in women with obstetric haemorrhage (in both low-income and high-income settings).^17 18^ Shock Index (pulse/systolic blood pressure) is a novel marker known to be an early predictor of complications in trauma/combat settings^19–23^ and a more consistent predictor of adverse outcomes than individual conventional vital signs (blood pressure, pulse, mean arterial pressure and pulse pressure) in obstetric haemorrhage.^17 18^ We confirmed Shock Index normal range in a prospective observational study.^24^ Shock Index thresholds were selected for incorporation into the traffic light early warning system according to associated risk of adverse outcomes (figure 2).^17 18^

## Methods

### Preliminary fieldwork

In 2013, we identified that over 90% of health clinics in a rural district of Tanzania either had no blood pressure devices or broken devices. We introduced 19 of our devices, containing tally counters to monitor use, into these clinics and showed that they were used frequently; focus groups confirmed they were well accepted by healthcare providers. This exploratory work led to a preintervention and postintervention study, conducted over 12 months in three rural hospitals in Tanzania, Zimbabwe and Zambia. During the 3-month preintervention phase, blood pressure was measured on consecutive pregnant women at 20 weeks’ gestation or more presenting to hospital for any reason. Blood pressure was measured twice (with a 5 min rest period between measurements) using the validated CRADLE prototype device (ie, without the traffic light early warning system) by trained volunteers. Urine dipstick results were recorded if available, together with demographic details and antenatal history, including whether blood pressure had ever been taken in the index pregnancy prior to presentation. These data were entered onto a central study database. During the 3-month intervention phase, 20 rural and semirural peripheral antenatal clinics that referred to each of the three hospitals received one to two of the CRADLE prototype devices (n=100) together with two face-to-face training sessions, translated instructions on its use and a guide on how to refer based on the blood pressure readings. One year later, in a matched time of year to the preintervention period, data collection was repeated as per the preintervention phase.

### Iterative qualitative evaluation

An iterative qualitative evaluation was undertaken to ensure that the VSA’s final traffic light early warning system and display symbols were understandable to users across a range of settings. Participants were shown a video animation of display options for hypertension and shock alerts and device utilities (low battery, error). Multiple choice questionnaires directly relating to the video animation and exploring the understanding of the options for the early warning system and the display symbols were distributed to a range of clinical and non-clinical participants from academic collaborative centres in India, the UK and Canada.

### Wide-range cuff validation

Incorrect cuff size leads to blood pressure measurement inaccuracies.^25–27^ All VSA device validations have included individuals with a range of arm circumferences, reflecting the general population. The VSA device comes with a medium cuff as standard and large cuffs as required. Obesity is a global problem, with prevalence reaching more than 70% in the Americas and Caribbean and more than 40% in Africa.^28^ In pregnancy, obesity is associated with increased risk of pre-eclampsia, obstetric haemorrhage and sepsis.^29–31^ To ensure accuracy in all women, particularly those with large arm circumferences, we endeavoured to validate the VSA device in combination with a novel wide-range cuff. This would also mean that healthcare providers would not need to estimate arm circumference and select the appropriate cuff prior to measurement.

The prospective validation study included 52 pregnant women recruited from a large tertiary hospital. Twenty-six women had an arm circumference of 22–32 cm and 26 had an arm circumference of 33–42 cm. Each woman had seven sequential same-arm measurements taken, alternating between mercury sphygmomanometer and the VSA prototype device and appropriate, inappropriate and wide-range cuffs. Data were analysed using adapted validation methods recommended by the BHS, the International Society of Hypertension (ISH) and the International Organization for Standardization (ISO) protocols.

### Auscultatory blood pressure validation

The VSA device can be used as an auscultatory device, using a stethoscope, and the falling digital display in a similar fashion to a mercury column while still providing a digital reading derived from oscillometry. This is particularly important in those with heart arrhythmias, as most automated devices fail to give accurate readings in these individuals, or when wanting to verify an unexpected automated reading. We undertook a prospective evaluation of clinician auscultatory VSA device measurement compared with VSA oscillometric measurement.

### Prospective clinical validation

A 15-month prospective validation of the early warning system of the VSA device was undertaken at three tertiary hospitals in South Africa. At the three maternity departments, existing blood pressure devices were replaced with VSA devices. Data, including vital signs and adverse clinical outcomes, were collected on all pregnant/postpartum women admitted with either pre-eclampsia, obstetric haemorrhage or sepsis. The performance of the traffic light early warning system thresholds to predict adverse clinical outcomes was evaluated.

## Results

### Preliminary fieldwork

Data from 1241 women were analysed (n=694 preintervention; n=547 postintervention). Introduction of the device to rural clinics was associated with improved antenatal surveillance of blood pressure (a decrease in the proportion of women who never had blood pressure measured in pregnancy from 25.1% to 16.9%, OR 0.58, p<0.001, 95% CI 0.42 to 0.79). This informed us that the device was acceptable to healthcare providers and may facilitate early treatment for women with pre-eclampsia.

### Iterative qualitative evaluation

Eighty-eight questionnaires were distributed and fully completed by doctors, nurses, midwives, healthcare assistants, medical students and non-clinical participants from India, the UK and Canada. Options not well understood were abandoned, including a blue traffic light to represent shock and an ‘E’ to represent an error. The options understood by the highest number of participants were selected for the final VSA device. With our team’s awareness that shock, particularly from haemorrhage, often requires more urgent intervention than hypertension, we ensured that the early warning system prioritised shock over hypertension and the shock alert demonstrated a higher level of urgency than hypertension (flashing down arrow as opposed to constant up arrow).

### Wide-range cuff validation

The wide-range cuff in combination with the VSA device failed validation in pregnant women with arm circumferences 22–42 cm, significantly overestimating both systolic and diastolic blood pressure in those with large arm circumferences, according to adapted BHS, ISH and ISO protocols. Therefore, the wide-range cuff cannot be recommended for use as a cuff suitable for a range of arm circumferences in a pregnant population. We went on to successfully validate the VSA device in combination with an extra-large cuff, meaning that the device is accurate when used in combination with medium, large and extra-large cuff sizes.

### Auscultatory blood pressure validation

Blood pressure readings (n=255) taken by 12 clinicians in the antenatal clinic, taking simultaneous and masked auscultatory and oscillometric readings, were analysed according to formal validation protocols. The mean difference (±SD) between clinician auscultation and oscillometry was 2.2 (±6.1) mm Hg and 2.3 (±5.2) mm Hg for systolic and diastolic blood pressure, respectively, well within the ISO protocol requirements (≤5±8 mm Hg). This confirmed that the VSA device maintains accuracy when used as an auscultatory device by trained healthcare providers.

### Prospective clinical validation

For obstetric haemorrhage and sepsis, the VSA traffic light thresholds strongly predicted risk of complications, including intensive care unit admission and emergency hysterectomy. For hypertension, the risk of renal impairment, preterm delivery, maternal magnesium sulfate use and intensive care unit admission increased with worsening VSA traffic light thresholds.^32^ The studies represent the first prospective cohort studies evaluating thresholds of blood pressure and pulse as predictors of adverse outcomes in pregnancy and the first to evaluate thresholds in LMICs.

## Discussion

### Current impact

To date (June 2018), over 6700 VSA devices have been used clinically in 12 countries across Africa, Asia and the Caribbean. An earlier version of the VSA device has been used as part of the intervention package in the community-level intervention for pre-eclampsia cluster randomised controlled trial in Mozambique, India and Pakistan, which enrolled a total of 75 532 pregnant women across the intervention and control clusters and completed recruitment in February 2017. In some cases, the VSA device was the first blood pressure device available to a clinic, and in most cases the first accurate blood pressure device for pregnancy in clinics and hospitals. In addition to the benefit to women in LMICs, the device is also suited for use in pregnancy in high-income countries, for non-pregnant adults and for home monitoring. Device accuracy when used as a self-monitor by pregnant women has been confirmed in a recent Danish prospective observational study.^33^ The device has also been adopted by groups requiring vital sign measurement away from clinical settings, including the fire brigade, army and mountain rescue in the UK.

We anticipate that women who would have previously gone unnoticed would now be identified early as being at risk. This simple technology may prevent maternal deaths and could contribute to meeting the United Nations Sustainable Development Goal 3: to reduce the global maternal mortality ratio to less than 70 000 per 100 000 live births by 2030. The potential contribution of the VSA device was recognised by the PATH Innovation Countdown 2030 Report, being nominated as one of 30 life-saving innovations with great promise to accelerate progress over the next 15 years to reach the Sustainable Development Goals.^34^

### Challenges and lessons learnt

It is recommended that all blood pressure devices are validated as accurate in the population for which they are intended.^13^ In 2006, although the VSA device passed non-pregnant adult validation, it concurrently failed validation in pregnancy, including pre-eclampsia. Over the next 5 years, the device’s algorithm was tweaked and revalidated. Only in 2012 did the device pass validation in pregnancy, including pre-eclampsia, highlighting the challenge of achieving accuracy in this particular population. This process delayed further device development and clinical evaluation but was crucial in ensuring a device that identifies abnormality and prevents morbidity.

An obstetric early warning system triggered by accurate blood pressure and pulse measurement is important for all settings. Maternity units in high-income countries frequently rely on non-validated blood pressure devices and early warning charts. Considering the high volume of women and frequency of blood pressure and pulse measurement in well-resourced tertiary units, the potential of a fully automated version of the device is being explored. The fully automated VSA device would be more suitable for well-resourced settings (and many LMIC facility-level settings), where power supply and cost are less of an issue. The current VSA device, with its low power requirements and low cost, would remain appropriate for low-resource settings.

### Where is the project heading next?

**Figure 3:**
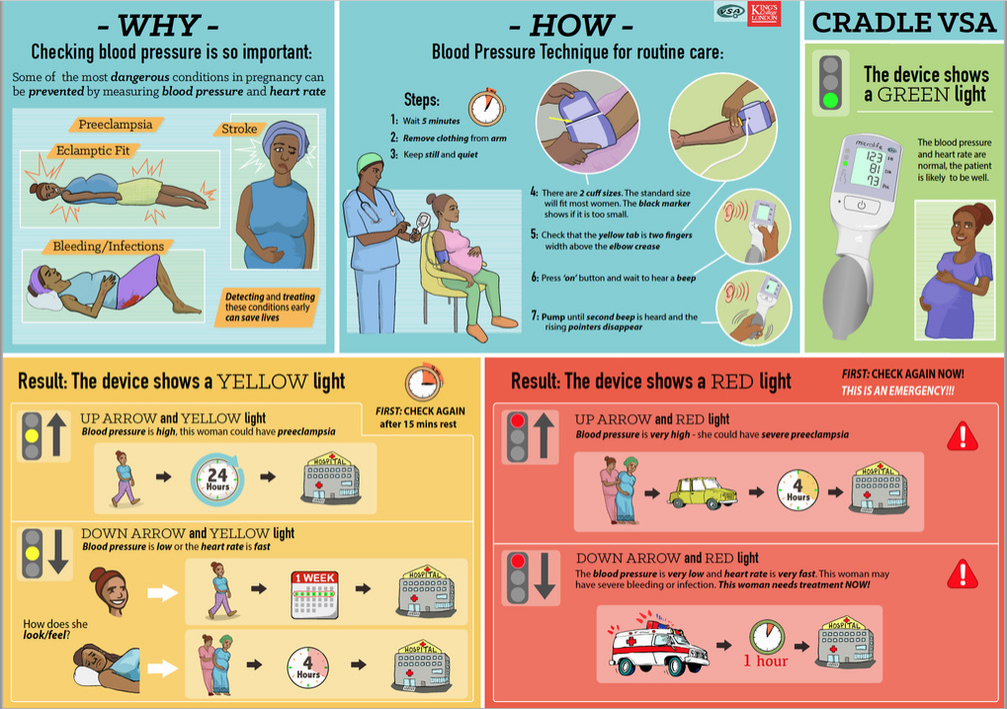
CRADLE trial training package poster for community healthcare providers. VSA, Vital Signs Alert.

A stepped-wedge, cluster randomised controlled trial (funded by the UK Medical Research Council/Department for International Development/Department of Biotechnology, India) to determine whether implementation of the CRADLE VSA device and a simple training package (figure 3) into every level of routine maternity care will reduce maternal mortality and morbidity in 10 LMIC sites (ISRCTN41244132) completed data collection in December 2017. The trial is guided by the Medical Research Council Complex Intervention Framework^35^ and commenced with a formative pilot phase where the training package was developed with multiple stakeholders input and then introduced, with the VSA device, into three LMIC sites. Feasibility and acceptability of the intervention and its implementation were assessed using questionnaires, observation, interviews and focus groups.^36^ This work informed the final training package, implementation strategies and data collection method for the trial. We will further determine the effectiveness of the CRADLE VSA intervention package and how the intervention may work in different contexts. The knowledge gained from this work will inform eventual scale-up.
